# Assessment of the Influence of Fabric Structure on Their Electro-Conductive Properties

**DOI:** 10.3390/ma17112692

**Published:** 2024-06-02

**Authors:** Magdalena Tokarska, Ayalew Gebremariam, Adam K. Puszkarz

**Affiliations:** 1 Institute of Architecture of Textiles, Faculty of Material Technologies and Textile Design, Lodz University of Technology, 116 Zeromskiego St., 90-543 Lodz, Poland; ayalew.gebremariam@dokt.p.lodz.pl; 2 Textile Institute, Faculty of Material Technologies and Textile Design, Lodz University of Technology, 116 Zeromskiego St., 90-543 Lodz, Poland; adam.puszkarz@p.lodz.pl

**Keywords:** electro-conductive fabrics, woven structure, van der Pauw method, electrical resistance, anisotropy, micro-computed tomography, smart textiles, wearable electronics

## Abstract

Electro-conductive fabrics are key materials for designing and developing wearable smart textiles. The properties of textile materials depend on the production method, the technique which leads to high conductivity, and the structure. The aim of the research work was to determine the factors affecting the electrical conductivity of woven fabrics and elucidate the mechanism of electric current conduction through this complex, aperiodic textile material. The chemical composition of the material surface was identified using scanning electron microscopy energy dispersion X-ray spectroscopy. The van der Pauw method was employed for multidirectional resistance measurements. The coefficient was determined for the assessment of the electrical anisotropy of woven fabrics. X-ray micro-computed tomography was used for 3D woven structure geometry analysis. The anisotropy coefficient enabled the classification of electro-conductive fabrics in terms of isotropic or anisotropic materials. It was found that the increase in weft density results in an increase in sample anisotropy. The rise in thread width can lead to smaller electrical in-plane anisotropy. The threads are unevenly distributed in woven fabric, and their widths are not constant, which is reflected in the anisotropy coefficient values depending on the electrode arrangement. The smaller the fabric area covered by four electrodes, the fewer factors leading to structure aperiodicity.

## 1. Introduction

Smart textiles are becoming increasingly popular in various fields such as wearable technology, sport and fitness, and military, medical, and aerospace applications [1,2,3]. Conductive textile materials are suitable for wearable electronics due to their high electrical conductivity. At the same time, these materials are characterized by small thickness and lightness [1,2,3,4] while maintaining flexibility and durability and providing heat and comfort for the user [5,6]. Electro-conductive textiles can be used for energy harvesting as a portable power supply system, textile signal lines for signal transmitting, and textile-based sensor production [3,7,8,9,10]. The development of innovative textile materials is connected with the individual yarn properties and integration methods of those yarns into fabrics such as weaving, knitting, and embroidery [10,11]. Electro-conductive threads incorporated into fabric can create electromagnetic interference shielding material to protect human health and sensitive electronic devices [12,13]. Metallization techniques enable the obtention of electrically conductive products from non-conductive textiles. As a result of surface modification, the sample is coated with thin conductive coating [14,15]. The density of threads in textile material is high, so the continuous coating is formed on the sample surface.

The anisotropic properties of materials encompassing ceramics [16], optical materials [17], composites [18], rocks [19], and textiles [5,20,21] are noticed and taken into consideration, particularly in engineering fields. The directional dependency of the property, such as thermal conductivity [22] or electrical resistance [23] of materials, is observed. The method widely used for measuring the electrical resistivity and carrier concentration is the van der Pauw technique [24]. It can be applied to semiconductors and metals [25,26,27]. This method enables the determination of surface resistance or resistivity of a thin material coating or layer [28,29]. The technique is beneficial for measuring the properties of irregularly shaped pieces and complex structures like flat textile products [20,30]. The van der Pauw method involves measuring the electrical resistance between four contacts placed on the edge of the sample, which is a two-dimensional geometry, typically a thin film or a plate. The van der Pauw electrode arrangement enables anisotropy detection in the sample plane [20,31,32], which is impossible [33] using the four-point probe method [34], where four electrodes are arranged collinearly on the sample surface. Two parameters are considered to be connected with the transverse and longitudinal axes to characterize the anisotropy of the material [23,35,36]. A quotient of electrical resistivities *ρ*_1_ and *ρ*_2_ associated with the two principal axes, wherein *ρ*_2_ ≥ *ρ*_1_, called the anisotropic coefficient, was used to assess the anisotropic electrical resistivities of the titanium films [35]. By applying a square root to the quotient, the modified coefficient was utilized in the electrical resistivity characterization of anisotropy in the aquifer [36]. The quotient in which the resistivities were replaced with resistances was used for the assessment of electro-conductive textile materials and called the biaxial anisotropy coefficient [23]. Based on the anisotropy measure defined as a quotient, it can be concluded that the material is isotropic if the coefficient equals one, which implies *ρ*_2_ = *ρ*_1_. A value above one means that the material exhibits anisotropy of the property.

The anisotropic electrical properties in the conductive woven fabric can be referred to as the difference in electrical resistance along the textile material warp, diagonal, and weft directions [23,31,33,37]. The anisotropy in electro-conductive textiles can be caused by the arrangement of conductive fibre and yarn configuration within the materials [37,38,39]. Thus, the multidirectional testing of textile materials is significant from the point of view of their electrical anisotropy and is not limited only to two principal axes in the sample plane, as indicated by literature reports [20,32,40,41]. The resistance of woven fabric is a crucial parameter when designing conductive fabric-based products. If the resistance value changes depending on the direction of testing, the electrical properties require in-depth analysis. Electro-conductive fabric as a measurement system component must work properly. An electro-conductive material intended for electrotherapy [10,39,42] with low electrical resistance and exhibiting isotropic properties can be connected to a stimulation device in any direction without the fear of resistance being too high and the current flow through the textile electrode causing growth in temperature and burn of the user’s skin. For an anisotropic fabric, it is possible to find a direction and method of connecting the sample to the measurement system so that higher resistance values are obtained. In the case of textile-based resistive sensors, detection of a wider operating range of resistance is expected to act as a pressure or damage detection sensor [1,9,43,44,45]. With a larger resistance range, the signal from the sensor is stronger and stands out clearer from background noise.

Depending on the application of electrically conductive fabrics, it is crucial to establish a test plan in which the multidirectional resistance measurements enable obtention of characteristics to complete sample analysis. The arrangement of the electrodes in the van der Pauw technique orients the sample with respect to the chosen direction in a plane [46]. This allows for the entire woven fabric to be considered during measurements and electrical planar anisotropy detection. Research was extended to include various variants of electrode arrangement. From the point of view of an electro-conductive fabric-based product designer, it is also essential to elucidate the mechanism of electric current conduction through this complex textile material. An analysis of electrical in-plane anisotropy in combination with the 3D geometry of the woven fabric structure is a new approach to understanding electrical conductivity in textile materials. A new anisotropy coefficient, considering electrical resistance multidirectional dependency, was determined for the evaluation and classification of fabric in terms of anisotropic and isotropic material. The research extends the knowledge of the phenomena of electric current conduction in fabrics.

## 2. Materials

**Figure 1:**
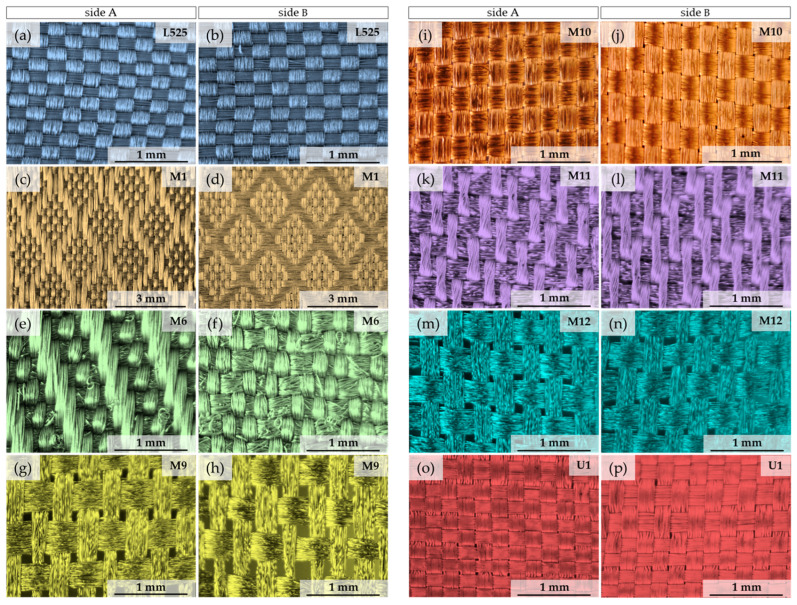
Optical images of tested woven fabrics: (**a**) side A of sample L525; (**b**) side B of sample L525; (**c**) side A of sample M1; (**d**) side B of sample M1; (**e**) side A of sample M6; (**f**) side B of sample M6; (**g**) side A of sample M9; (**h**) side B of sample M9; (**i**) side A of sample M10; (**j**) side B of sample M10; (**k**) side A of sample M11; (**l**) side B of sample M11; (**m**) side A of sample M12; (**n**) side B of sample M12; (**o**) side A of sample U1; (**p**) side B of sample U1.

The research and analysis of electrical in-plane anisotropy of conductive textile materials was conducted on eight commercially available woven fabrics, as depicted in Figure 1. There were no signs of any damage or surface defects in the samples. The available information about fabrics and the basic parameters of the textile materials are given in Table 1. Measurements of fabric thickness and fabric mass were repeated five times.

The chemical composition analysis of the fabric surface was conducted using a scanning electron microscope energy dispersion X-ray spectroscopy (SEM-EDS) method [47,48,49]. The identification of the amount of the main elements on the fabric surface was carried out to confirm the occurrence of metals. The JEOL JSM-6610 LV microscope (Jeol, Tokyo, Japan) equipped with an X-MAX 80 module (Oxford Instruments, High Wycombe, UK) was used. The weight percentage (wt%) of the main chemical elements responsible for sample conductivity was determined and presented in Table 2; the word “Others” denotes the remaining chemical elements (Si, Cl, C, O, P). The standard deviation SD of all elements weight percentages was estimated at 0.1 wt%.

The data presented in Table 2 show that the elemental composition of the samples differs. Sample L525 has a thin Cu-Ni coating (25.4 wt%) because the combined content of the other elements is higher than that of the remaining fabrics. Samples U1, M11, and M10 have thick coating, and the combined content of the other elements is lower than that of the remaining fabrics.

**Figure 2:**
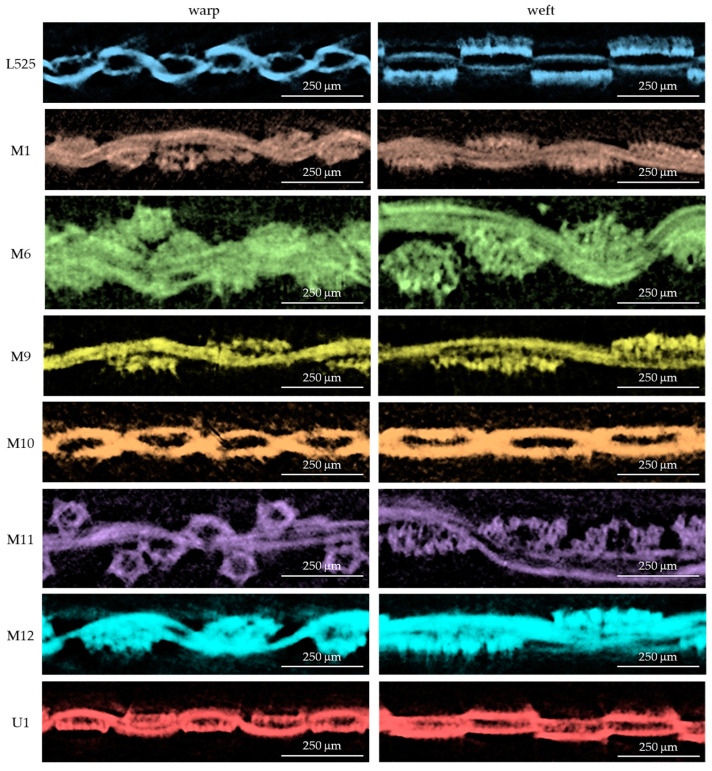
Micro-CT cross-sections of tested fabrics.

X-ray micro-computed tomography, micro-CT (SkyScan 1272, Bruker, Kontich, Belgium) [50,51], was used to determine the geometrical parameters of 3D woven fabrics: weave type, warp and weft densities, and their elliptical cross-section. The major axis of the elliptical warp/weft cross-section is associated with the fabric plane (width and length). The minor axis of the cross-section is related to fabric thickness. The geometrical parameters are given in Table 3. The measurements were repeated three times. The cross-sections of tested fabrics (along the warp and weft) are presented in Figure 2.

## 3. Methods

**Figure 3:**
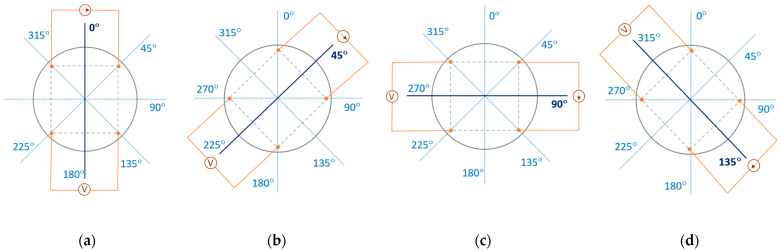
Van der Pauw configuration of electrodes on the circular sample for the direction defined by angle θ in the sample plane: (**a**) θ = 0°; (**b**) θ = 45°; (**c**) θ = 90°; (**d**) θ = 135°.

The study on the electrical anisotropy of conductive woven fabrics was based on the four-point probe method with van der Pauw electrode configuration. Eight electro-conductive fabrics were used to prepare circular sample fabrics with a 10-centimetre diameter (ϕ = 10 cm). Four electrodes were placed on the sample surface, forming a square (Figure 3). The cylindrical brass electrodes were used. The contact area of the electrode with the textile substrate was 2 mm in diameter. Two upper electrodes were connected to the direct current (DC) source (Agilent E3644A, Agilent Technologies, Santa Clara, CA, USA), and the two bottom electrodes were connected to the voltmeter (Agilent 34410A, Agilent Technologies, USA). Four electrodes were simultaneously rotated clockwise at 45° starting from the initial position (Figure 3a). The endpoint corresponded to θ = 360°. Two electrical resistance measurements per direction defined by pairs of the following angles θ were obtained: 0° and 180° (Figure 3a), 45° and 225° (Figure 3b), 90° and 270° (Figure 3c), 135° and 315° (Figure 3d). Each pair means exchanging voltage and current electrodes.

**Figure 4:**
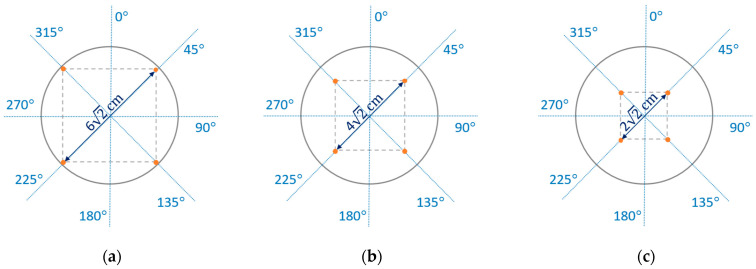
Arrangement of electrodes on sample plane: (**a**) L; (**b**) M; (**c**) S.

Three possibilities for arranging the electrodes, L, M, and S, on a fabric surface were considered wherein four electrodes form squares with side lengths of 6 cm, 4 cm, and 2 cm, respectively (Figure 4).

**Figure 5:**
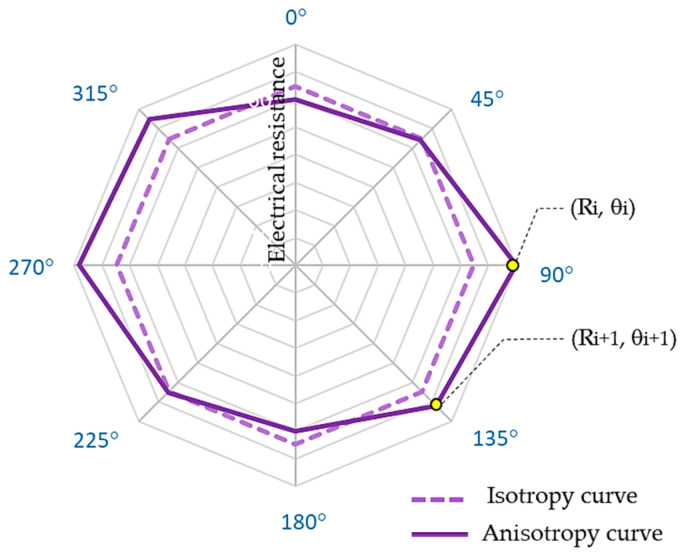
Curves representing electrical isotropy and anisotropy of fabric.

The test samples were used to find the minimum voltage drop value, and the L arrangement of electrodes was applied to establish the starting point (Figure 3a) on the sample plane. A direct current was equal to 0.040 A to avoid the electrode and sample heating effect. Voltage drop measurements on woven fabrics were conducted for all arrangements of electrodes, i.e., L, M, and S, assuming the same starting point. Electrical resistance was calculated according to Ohm’s law. The resistance values were expected to be the same for isotropic fabric samples in every direction. Electrical in-plane anisotropy manifests in variable resistance values R depending on the testing direction θ. The anisotropy of fabric can be presented as an anisotropy curve. A schematic comparison of the isotropy and anisotropy curves is shown in Figure 5.

We let (R_i_,θ_i_), (R_i+1_,θ_i+1_) are neighbouring two-dimensional polar coordinates of points belonging to the anisotropy curve (Figure 5) where θ_i+1_ − θ_i_ > 0, i = 1, 2, …, n, and n is the number of segments of the anisotropy curve. The length of the anisotropy curve is given as follows:

(1) $$D_{aniso}=\sum_{i=1}^{n}\sqrt{R_{i}^{2}+R_{i+1}^{2}-2R_{i}R_{i+1}\cos\left(\theta_{i+1}-\theta_{i}\right)}$$

We let angle θ, be changed from 0° to 360°. In the case of the isotropic sample, we obtain R_i+1_ = R_i_ = R_m_ for i = 1, 2, …, n. Using Equation (1), we obtain the length of the isotropy curve:

(2) $$D_{iso}=R_{m}\sum_{i=1}^{n}\sqrt{2-2\cos\left(\theta_{i+1}-\theta_{i}\right)}$$

where, for anisotropic samples, the mean resistance value can be expressed by formula [20,52,53]

(3) $$R_{m}=\sqrt[{n}]{\prod_{i=1}^{n}R_{i}}$$

Assuming the constant angle increment θ_i+1_ − θ_i_ = 45° ∀i=1,2,…,n (n = 8), the length of the isotropy curve is expressed as

(4) $$D_{iso}=8R_{m}\sqrt{2-\sqrt{2}}$$

Parameter D% is adopted to measure the electrical in-plane anisotropy of conductive woven fabric, which determines the relative difference in the length of the anisotropy and isotropy curves. The anisotropy coefficient D% is given as follows:

(5) $$D\%=\frac{D_{aniso}-D_{iso}}{D_{iso}}100\%$$

A value of D% equal to zero indicates that the fabric exhibits isotropic electrical properties. The higher the value of the anisotropy coefficient, the higher the electrical in-plane anisotropy of the fabric.

## 4. Results and Discussion

### 4.1. Electrical Anisotropy of Fabrics

**Figure 6:**
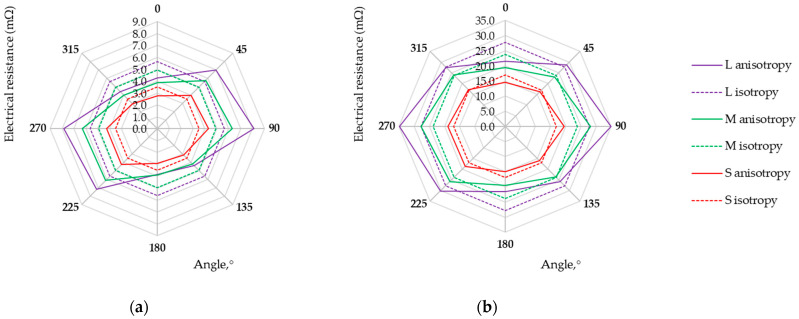
Isotropy and anisotropy curves for samples (**a**) L525; (**b**) M1.

**Figure 7:**
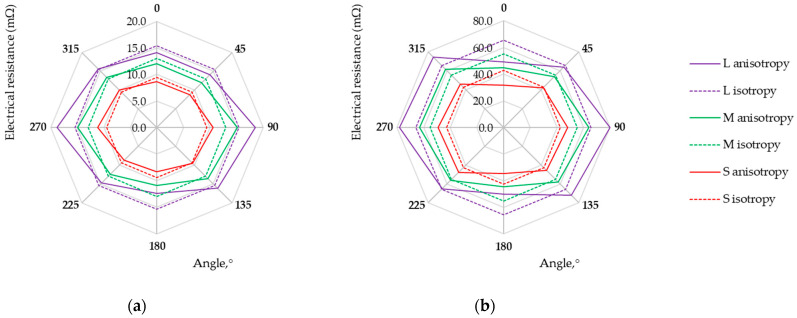
Isotropy and anisotropy curves for samples (**a**) M6; (**b**) M9.

**Figure 8:**
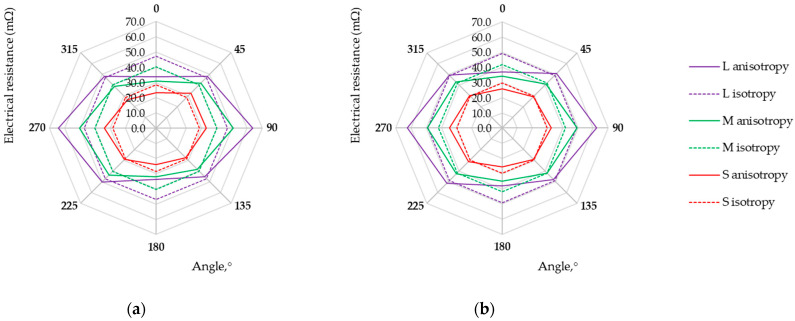
Isotropy and anisotropy curves for samples (**a**) M10; (**b**) M11.

**Figure 9:**
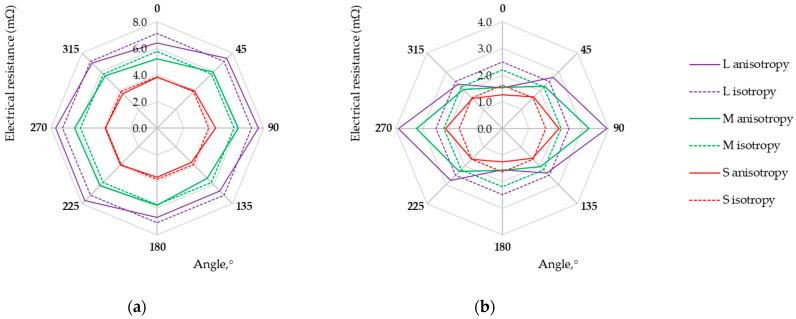
Isotropy and anisotropy curves for samples (**a**) M12; (**b**) U1.

Measurements of the voltage drop for a fixed current value for electro-conductive fabrics were carried out at a temperature of 22 °C and relative humidity of 39%. Each measurement was repeated five times. The direction-dependent resistance of electro-conductive fabrics in the form of isotropy and anisotropy curves is presented in Figure 6, Figure 7, Figure 8 and Figure 9. Three possibilities for arranging the electrodes, L, M, and S, were considered.

Research results showed that the maximum and minimum resistance values are related to the direction of the warp and weft threads, regardless of the fabric weave. Except for the anisotropy curves for three arrangements of electrodes (L, M, and S), the isotropy curves were also determined based on the geometric mean R_m_ (Equation (3), see Table 4) and the results are presented in Figure 6, Figure 7, Figure 8 and Figure 9.

The length of the isotropy curve (Equation (2)) and the anisotropy curve (Equation (1)) were determined. Finally, the anisotropy coefficient D% was calculated (Equation (5)) to assess the electrical in-plane anisotropy of conductive woven fabrics. The determined characteristics are given in Table 4.

The anisotropy coefficient values range from 0.7% to 9.9%, 0.8% to 13.5%, and 0.9% to 20.5% for the S, M, and L arrangement of electrodes, respectively. This indicates that the electrical conductivity of fabrics varies depending on the direction determined in the fabric plane. The woven structure consists of reports repeated throughout the length and width of the fabric. Thus, the structure of an ideal woven fabric is periodic. The larger the fabric area covered by four electrodes (S→M→L), the more threads and contacts there are between them. The threads in the actual fabric are unevenly distributed, their widths are not constant, and the conductive coating on the fabric surface differs. Therefore, the smaller the surface (L→M→S), the fewer disturbances and the decrease in the anisotropy coefficient D% for each woven fabric.

The assessment of woven fabrics in terms of isotropic or anisotropic material based on the D% value is not obvious. The qualification of the sample depends on the material used and the requirements for the textile-based product. An anisotropy coefficient of several percent may be unacceptable for medical applications but acceptable for a material intended for use in industrial conditions. The final decision is up to the researcher/designer. Fabric M12, which can be treated as isotropic from the point of view of electrical conductivity (see Figure 9a), can be used as the electrode for muscle electrostimulation. Fabric M12, acting as an electrode, can be employed in various orientations without concern for directional conductivity. Small values of fabric M12 resistance R_m_ (at a level not exceeding 7 mΩ) and uniform electrical conductivity (D% below 1%) should help prevent potential skin irritation or burns that could result from the uneven current distribution. Fabrics exhibiting the highest electrical anisotropy, L525 and U1 from the tested fabrics, may be suitable for applications where directional conductivity is advantageous. A higher resistance leads to designing a sensor that can detect the smaller variations in the measured quantity, consequently creating more sensor sensitivity. In the case of fabrics L525 and U1, the mean resistance value does not exceed 6 mΩ and 3 mΩ, respectively. Fabrics M1, M6, M9, M10, and M11 seem to be better from this point of view, wherein the highest values of resistances R_m_ characterize M9, M10, and M11. A fabric-based sensor should be connected to the measuring system in such a way as to obtain the highest possible fabric resistance value. Studies of the multidirectional dependency of electrical resistance in the sample plane provide this knowledge. For considered fabrics M9, M10, and M11, the resistance values depending on the testing direction are shown in Figure 7b and Figure 8.

### 4.2. Factors Affecting the Electrical Conductivity of Fabrics

Analysis of the impact of the electrode arrangement (L, M, and S) on the resistance values (R_L_, R_M_, and R_S_, respectively) obtained for angles θ_i_ (i = 1, 2, …, n) was carried out based on the Kruskal–Wallis test (K-W test) [54]. The test leads to significant results at the assumed significance level when at least one group differs from the others. A significance level of 0.05 was assumed. The testing of the null hypothesis H_0_: R_L_ = R_M_ = R_S_ against the alternative hypothesis H_1_: R_L_ ≠ R_M_ ≠ R_S_ is conduced through the *p*-value method. If the *p*-value is less than 0.05, there are significant differences in groups at a 0.05 significance level; if the *p*-value is greater than 0.05, there are no significant differences. The *p*-values were determined using TIBCO Statistica 13.3 software. A post hoc test was used to identify groups L-M, M-S, and L-S, which differ significantly. Results of the K-W and post hoc tests are presented in Table 5. The significant differences in values of resistances in groups for all samples except U1 were found based on the K-W test.

It was noticed that there were no significant differences in values of resistances obtained for the arrangement of electrodes L and M (group L-M) for all the tested woven fabrics. Significant differences were observed in group M-S for some fabrics (M1, M6, M10, and M11). It was stated that there were significant differences in values of resistances between L and S arrangements of electrodes (group L-S) for all fabrics besides U1. Thus, it was stated that the number of interlaced threads in the woven fabric resulting from the area size defined by four electrodes is a factor that can significantly impact resistance measurements.

The correlation analysis was conducted to discover whether there is a linear relationship between pairs of chosen variables (factors). The values of Pearson’s correlation coefficients are given in Table 6. The significant correlation coefficients at a 0.05 significance level are marked in red.

It was found (see Table 6, cells marked in yellow) that the values of the coefficients of anisotropy D% increased with the increase in the distance between the electrodes. The mean value of resistance R_m_ also increased in these conditions (see Table 6, cells marked in orange). A strong positive correlation was noticed in both cases. According to Ohm’s law, assuming the conductor has the same cross-sectional area, its resistance increases as its length (the distance between the electrodes) increases. Correlation between the values of resistances R_m_ obtained for different arrangements of electrodes (L, M, and S) and structural parameters such as the weft/warp density (DEwe, DEwa) and the major axis of elliptical weft/warp cross-section (MAwe, MAwa) was not found (see Table 6, cells marked in grey) for the tested fabrics. The analysis also indicated no correlation between the mean resistance of fabrics and the amount of the main electro-conductive chemical elements detected and identified on the sample surface (see Table 6, cells marked in blue). The coating containing metals of very low resistivity (Cu, Ni, Ag—the resistivity of order 10^−8^ Ωm, and Sn 10^−7^ Ωm, at 20 °C) ensured good electrical conductivity for the textile materials (L525, M9, M10, M11, M12, U1). The electro-conductive coating is so thin that it does not create a smooth metallic layer on the textile substrate; the features of the woven structure are preserved. The multidirectional fabric resistance measurements indicated that the surface structure features play a significant role in electric current conduction. This fact can be especially true in the case of M1 and M6 fabrics which, according to the information provided by the supplier (Table 1), are woven from silver-plated polyamide yarns and not metalized like the remaining textile materials.

Strong positive correlations were found between the coefficients of anisotropy (case of L and M electrode arrangements) and the density of weft (see Table 6, cells marked in purple). The increase in the number of threads causes an increase in the number of contacts between threads. The surface complexity impacts the electrical anisotropy of the fabric. Moreover, the fabric is not an ideal periodic woven structure, as indicated by the standard deviations of the parameters in Table 3. Values of standard deviations may reach a dozen percent of the average value of the geometrical parameter. It was noticed that coefficient D% decreases with the rise in the length of the major axis of the elliptical weft cross-section (see Table 6, cells marked in green). The major axis of the thread, which is its width measure, is a parameter responsible for flattening the fabric. The width of the conductor, which can be the thread, matters; a wider conductor has lower resistance than a narrower one with the same cross-section. The identification of the shape of the elliptical cross-section of the yarn is indirectly the identification of the cross-section of the conductive path in the fabric, assuming the depth of electric current penetration is the same. It was found that the major axis of warp decreases when the number of warp and weft threads increase (see Table 6, cells marked in pink), which results from the weaving process.

## 5. Conclusions

Determining the multidirectional dependency of electrical resistance allows for evaluating the planar anisotropy of woven fabrics. The anisotropy coefficient enables the classification of electro-conductive fabrics in terms of isotropic or anisotropic materials. The coefficient advantage is that it considers the resistance values specified for all chosen directions, not just the two connected with principal axes. It can be concluded that woven structures are anisotropic rather than isotropic materials. It was found that the increase in weft density results in an increase in the anisotropy of the sample. The rise in thread width can lead to smaller anisotropy of woven fabric. Based on micro-CT analysis of 3D fabric geometry, it was found that threads are unevenly distributed in woven fabric, and their widths are not constant throughout the length and width of the sample. This is reflected in the anisotropy coefficient values depending on electrode arrangement. The smaller the fabric area covered by four electrodes (L→M→S), the fewer disturbances there are and the lesser decrease in the anisotropy coefficient D% for each fabric.

## Figures and Tables

**Table 1:** Characteristics of electrically conductive fabrics.

Sample	Raw Material Composition (Supplier)	Electrical Resistance	Thickness ± SD ^1^ (mm)	Surface Mass (g m^−2^)
L525	Copper and nickel metalized polyamide fabric (Laird)	<0.07 Ω	0.12 ± 0.01	86
M1	Silver-coated polyamide yarns (Qingdao Hengtong X-Silver)	Conductive	0.15 ± 0.01	84
M6	Silver-coated polyamide yarns (Qingdao Hengtong X-Silver)	Conductive	0.35 ± 0.01	164
M9	Nickel metalized polyester fabric (Soliani EMC)	<0.40 Ω	0.19 ± 0.01	65
M10	Nickel metalized polyester fabric (Soliani EMC)	<0.40 Ω	0.08 ± 0.01	75
M11	Nickel metalized polyester fabric (Soliani EMC)	<0.40 Ω	0.27 ± 0.01	152
M12	Nickel metalized polyester fabric (Soliani EMC)	<0.40 Ω	0.15 ± 0.02	113
U1	Copper and tin metalized polyamide fabric (Shieldex)	<0.02 Ω	0.12 ± 0.01	77

^1^ SD—standard deviation.

**Table 2:** Fabric surface chemical composition ^2^.

Chemical Element→ Sample ↓	Cu	Ni	Ag	Sn	P	Ti	Others
L525	12.1 ± 0.1	13.4 ± 0.1	-	-	-	-	74.6
M1		-	53.0 ± 0.1	-	-	-	47.0
M6	-	-	62.9 ± 0.1	-	-	-	37.1
M9	-	49.1 ± 0.1	-	-	0.6 ± 0.1	0.1 ± 0.1	50.2
M10		82.7 ± 0.1			1.7 ± 0.1		15.6
M11		87.4 ± 0.1			0.6 ± 0.1		12.0
M12	-	60.6 ± 0.1	-	-	0.1 ± 0.1	-	39.3
U1	76.7 ± 0.1	-	0.3 ± 0.1	12.5 ± 0.1	-	-	10.5

^2^ The amount of elements is expressed in wt%; the standard deviation is also given.

**Table 3:** Geometrical structure of the fabrics ^3^.

Sample Parameter	L525	M1	M6	M9	M10	M11	M12	U1
**Weave**	Twill	Dobby	Twill	Plain	Plain	Twill	Plain	Ripstop
**Weft density (mm^−1^)**	4.2 ± 0.11	3.1 ± 0.15	3.5 ± 0.094	1.9 ± 0.13	3.2 ± 0.099	3.6 ± 0.11	2.4 ± 0.079	4.6 ± 0.14
**Warp density (mm^−1^)**	6.3 ± 0.088	4.6 ± 0.18	7.3 ± 0.13	3.2 ± 0.20	4.2 ± 0.084	6.3 ± 0.15	3.2 ± 0.087	5.2 ± 0.18
**Major axis of elliptical weft cross-section (mm)**	0.24 ± 0.030	0.29 ± 0.051	0.25 ± 0.041	0.26 ± 0.035	0.29 ± 0.047	0.25 ± 0.065	0.31 ± 0.048	0.22 ± 0.052
**Minor axis of elliptical weft cross-section (mm)**	0.09 ± 0.004	0.08 ± 0.005	0.1 ± 0.009	0.08 ± 0.008	0.08 ± 0.007	0.1 ± 0.005	0.07 ± 0.007	0.05 ± 0.004
**Major axis of elliptical warp cross-section (mm)**	0.13 ± 0.023	0.19 ± 0.043	0.11 ± 0.021	0.35 ± 0.056	0.22 ± 0.045	0.14 ± 0.025	0.24 ± 0.059	0.16 ± 0.051
**Minor axis of elliptical warp cross-section (mm)**	0.04 ± 0.003	0.08 ± 0.004	0.09 ± 0.004	0.05 ± 0.003	0.05 ± 0.003	0.07 ± 0.004	0.08 ± 0.005	0.05 ± 0.003

^3^ Standard deviation is additionally given.

**Table 4:** Anisotropy characteristics of tested fabrics.

Sample	Electrode Arrangement	R_m_ (mΩ) (GSD) ^4^	D_iso_ (mΩ)	D_aniso_ (mΩ)	D%
L525	L	5.63 (1.4)	34.47	39.91	15.8%
	M	4.94 (1.3)	30.24	32.91	8.8%
	S	3.51 (1.2)	21.49	22.84	6.3%
M1	L	27.73 (1.2)	169.81	179.84	5.9%
	M	23.75 (1.2)	145.45	150.26	3.3%
	S	16.91 (1.1)	103.56	105.81	2.2%
M6	L	15.43 (1.2)	94.45	97.82	3.6%
	M	12.97 (1.1)	79.40	81.27	2.4%
	S	9.42 (1.1)	57.68	58.84	2.0%
M9	L	65.63 (1.2)	401.87	425.69	5.9%
	M	55.24 (1.2)	338.25	350.07	3.5%
	S	42.68 (1.2)	261.35	273.03	4.5%
M10	L	47.05 (1.3)	288.11	316.06	9.7%
	M	40.17 (1.2)	245.97	260.07	5.7%
	S	28.49 (1.2)	174.42	180.72	3.6%
M11	L	49.04 (1.2)	300.26	318.68	6.1%
	M	41.81 (1.1)	255.98	263.82	3.1%
	S	29.64 (1.1)	181.46	184.81	1.8%
M12	L	7.11 (1.1)	43.52	43.92	0.9%
	M	5.76 (1.1)	35.29	35.57	0.8%
	S	3.86 (1.1)	23.65	23.81	0.7%
U1	L	2.49 (1.4)	15.28	18.42	20.5%
	M	2.20 (1.3)	13.47	15.29	13.5%
	S	1.63 (1.2)	9.96	10.65	9.9%

^4^ GSD—geometric standard deviation.

**Table 5:** The *p*-values as the results of Kruskal–Wallis and post hoc tests ^5^.

Sample	K-W Test	Post Hoc Test		
		L-M	M-S	L-S
L525	**0.0141**	0.7088	0.2592	**0.0112**
M1	**0.0003**	0.7962	**0.0148**	**0.0003**
M6	**0.0002**	0.3853	**0.0296**	**0.0001**
M9	**0.0012**	0.2496	0.1555	**0.0007**
M10	**0.0017**	0.9666	**0.0400**	**0.0016**
M11	**0.0003**	0.7300	**0.0175**	**0.0003**
M12	**0.0000**	0.0709	0.0709	**0.0000**
U1	0.0650	1.0000	0.3853	0.0647

^5^ Values indicating significant differences are in bold.

**Table 6:** Matrix with correlation coefficients for chosen factor comparison ^6^.

	DEwe	DEwa	MAwe	MAwa	eChE	Rm L	Rm M	Rm S	D% L	D% M	D% S
**DEwe**	1.0000	0.7280	−0.6897	−0.8513	0.2004	−0.5942	−0.5868	−0.6050	0.7589	0.7471	0.5335
**DEwa**	0.7280	1.0000	−0.6084	−0.8995	0.0158	−0.3252	−0.3201	−0.3378	0.2399	0.2028	0.1158
**MAwe**	−0.6897	−0.6084	1.0000	0.4379	−0.0605	0.1961	0.1949	0.1748	−0.7120	−0.7076	−0.7269
**MAwa**	−0.8513	−0.8995	0.4379	1.0000	−0.1346	0.5837	0.5766	0.6078	−0.3130	−0.2921	−0.0618
**eChE**	0.2004	0.0158	−0.0605	−0.1346	1.0000	0.1777	0.1814	0.1504	0.0878	0.1457	−0.1304
**Rm L**	−0.5942	−0.3252	0.1961	0.5837	0.1777	1.0000	0.9999	0.9985	−0.3414	−0.3811	−0.1816
**Rm M**	−0.5868	−0.3201	0.1949	0.5766	0.1814	0.9999	1.0000	0.9980	−0.3364	−0.3770	−0.1799
**Rm S**	−0.6050	−0.3378	0.1748	0.6078	0.1504	0.9985	0.9980	1.0000	−0.3296	−0.3677	−0.1538
**D% L**	0.7589	0.2399	−0.7120	−0.3130	0.0878	−0.3414	−0.3364	−0.3296	1.0000	0.9913	0.9278
**D% M**	0.7471	0.2028	−0.7076	−0.2921	0.1457	−0.3811	−0.3770	−0.3677	0.9913	1.0000	0.9157
**D% S**	0.5335	0.1158	−0.7269	−0.0618	−0.1304	−0.1816	−0.1799	−0.1538	0.9278	0.9157	1.0000

^6^ DEwe/DEwa—weft/warp density, MAwe/MAwa—major axis of elliptical weft/warp cross-section, eChE—electro-conductive chemical elements.

## Data Availability

The raw data supporting the conclusions of this article will be made available by the authors on request.
